# Contrasted phylogeographic patterns on mitochondrial DNA of shallow and deep brittle stars across the Atlantic-Mediterranean area

**DOI:** 10.1038/srep32425

**Published:** 2016-09-02

**Authors:** Sergi Taboada, Rocío Pérez-Portela

**Affiliations:** 1 Department of Life Sciences, The Natural History Museum of London, Cromwell Road, SW7 5BD, UK; 2 Centro de Estudios Avanzados de Blanes, CSIC, Accés a la cala St. Francesc, 14, 17300, Blanes, Spain

## Abstract

Previous studies on *Ophiothrix* in European waters demonstrated the existence of two distinct species, *Ophiothrix fragilis* and *Ophiothrix* sp. II. Using phylogenetic and species delimitation techniques based on two mitochondrial genes (cytochrome *c* oxidase I and *16S rRNA*) we prove the existence of a new congeneric species (*Ophiothrix* sp. III), occurring in the deep Atlantic coast of the Iberian Peninsula and the Alboran Sea. We compared phylogeographic patterns of these three *Ophiothrix* species to test whether closely related species are differentially affected by past demographic events and current oceanographic barriers. We used 432 sequences (137 of *O. fragilis*, 215 of *Ophiothrix* sp. II, and 80 of *Ophiothrix* sp. III) of the *16S rRNA* from 23 Atlantic-Mediterranean locations for the analyses. We observed different geographic and bathymetric distributions, and contrasted phylogeography among species. *Ophiothrix fragilis* appeared genetically isolated between the Atlantic and Mediterranean basins, attributed to past vicariance during Pleistocene glaciations and a secondary contact associated to demographic expansion. This contrasts with the panmixia observed in *Ophiothrix* sp. II across the Atlantic-Mediterranean area. Results were not conclusive for *Ophiothrix* sp. III due to the lack of a more complete sampling within the Mediterranean Sea.

Within the past few decades, phylogeographic patterns in marine benthic invertebrates with Atlantic-Mediterranean distribution have been documented in a plethora of species belonging to a range of phyla *e.g*. refs 1, 2, 3, 4, 5, 6, 7, 8, 9. The relatively well documented geological history of the Mediterranean Sea and its connection with the Atlantic Ocean has allowed in most cases relating historical processes (*e.g.* the Messinian Salinity Crisis –MSC– during Mio-Pliocene transition, and more recently, the Pleistocene glacial and interglacial periods during the Quaternary), to the current population connectivity patterns observed for different species *e.g.* refs 3, 10, 11, 12, 13, 14, 15, 16. Apart from these historical processes, the current hydrography, including major circulation patterns and local eddies, and geographical processes differentially affect the littoral areas from both the Atlantic and Mediterranean basins. These geographical, historical and oceanographic processes, altogether with the inherent biological characteristics of the species, are among the responsible processes for the present-day intraspecific distribution of genetic diversity in littoral species^1^,^17^. Nevertheless, the stochasticity of the recruitment processes and availability of substrate can also play an important role shaping the current genetic structure of benthic taxa^18^.

The Strait of Gibraltar represents a natural connection, and at the same time a transition area, between the different hydrographical conditions that go from the relatively cold waters of the North Atlantic to the warmer, saltier, and markedly seasonal waters of the Mediterranean^19^. However, the Alboran Sea and more specifically the Almeria-Oran Front (AOF), situated east of the Strait of Gibraltar, is considered as the real biogeographical break between these two basins. A number of marine species show genetic transition between Atlantic and Mediterranean populations at the AOF, which acts as a migration barrier preventing or significantly reducing genetic flow between these two areas (see a review in Patarnello *et al.*^1^, but see García-Merchán *et al.*^7^). Genetic connectivity between populations of these two basins including the AOF as a target area has been addressed for several benthic invertebrates, reaching in some cases different conclusions even when considering species with similar dispersal abilities *e.g*. refs 7, 8, 12 and 20, which may be explained by the effect of past historical processes differentially affecting the genetic diversity of these organisms. In this sense, the comprehensive study of congeneric species with a common evolutionary origin (*i.e.* sharing similar biological traits) and similar geographic distribution (*i.e.* being affected by similar hydrological and geological processes), has been proposed as a useful approach to identify phylogeographic signals and real permeability of major marine fronts^1^,^7^,^21^.

Due to their wide geographic distribution, extending from the intertidal to the deep subtidal^22^, and their ecological relevance, brittle stars of the genus *Ophiothrix* appear as an interesting study case to address questions about connectivity among populations occurring in both the Atlantic and Mediterranean basins, as well as potential processes of cryptic speciation and multi-specific complexes. In an investigation of *Ophiothrix fragilis* on different intertidal and subtidal Atlantic populations, also including a few Western Mediterranean specimens, Muths *et al.*^23^ identified two distinct lineages that might potentially correspond to cryptic species. One of these lineages corresponded to the northern European populations, and another one encompassed specimens from two sites of the south European coast: one at the Atlantic coast of the Iberian Peninsula, in the intertidal of Galicia, and another from a subtidal site at the French Coast of Banyuls-sur-mer (NW-Mediterranean). Later, Pérez-Portela *et al.*^15^ conducted an extensive study combining molecular and morphological analyses using populations from both the Atlantic and the Mediterranean basins, and concluded that the two different lineages identified by Muths *et al.*^23^ in fact corresponded to two different morphological species (Lineage I: *O. fragilis*, and Lineage II: *Ophiothrix* sp. II). One of the species identified, *O. fragilis,* inhabits the intertidal and shallow subtidal (0–50 m) in the North Atlantic, where it occurs in highly dense assemblages^22^, but it also occurs in the Mediterranean deep subtidal (>50 m), where it was previously described as *O. quinquemaculata* (see ref. 15). Unfortunately, the study by Pérez-Portela *et al.*^15^ only included two deep subtidal Mediterranean samples (~50–60 m), and sequences of the so-called *O. quinquemaculata* from Genbank; therefore conclusions about the distribution of *O. fragilis* along the Mediterranean area were very limited.

In the study by Pérez-Portela *et al.*^15^, shallow-water populations of *Ophiothrix* sp. II across the Atlantic and Mediterranean showed apparent panmixia with no gene-flow barriers across the study area. This pattern was explained by a combination of processes including a wide dispersal potential, large effective population size and a recent demographic expansion. On the other hand, information on the genetic structure between populations of *O. fragilis* in the Atlantic area, along the coast of France and England, showed a chaotic structure in this species^23^. Genetic connectivity between Atlantic individuals and the only individual analysed from the Western Mediterranean, though, were not conclusive and pointed to future studies to unravel the genetic connectivity of this species.

Here, we present a comprehensive genetic study in *Ophiothrix* spp. occurring in the Atlantic and Mediterranean basins covering from the intertidal to the shallow and deep subtidal. The aims of this work were: (i) to describe the distribution of *Ophiothrix* spp. across both space and depth, and to detect the possible occurrence of cryptic speciation; and (ii) to compare phylogeographic patterns based on mitochondrial DNA among congeneric species to determine whether present and past marine biogeographic barriers and demographic events may have differentially affected the distribution of genetic diversity among species, paying special attention to potential differentiation between the Atlantic and Mediterranean basins. To achieve our objectives we used two mitochondrial gene fragments, the *16S rRNA* (hereafter *16S*), which has been demonstrated to provide suitable resolution in population genetic studies of brittle stars^15^,^24^, and the cytochrome *c* oxidase subunit I (hereafter *COI*).

## Results

### Phylogeny and species delimitation

**Figure 1:**
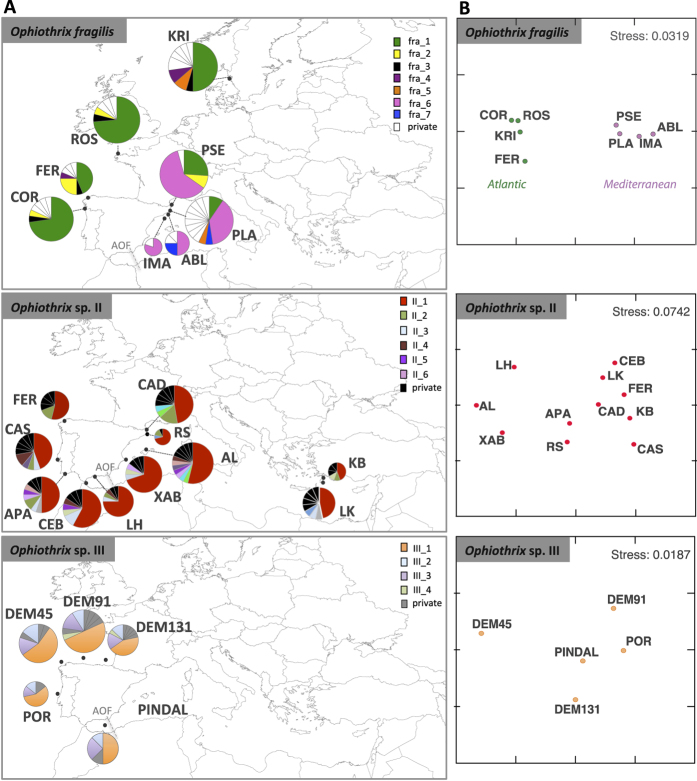
(**A**) Map of the Atlantic-Mediterranean area showing the locations where samples of *Ophiothrix fragilis*, *Ophiothrix* sp. II, and *Ophiothrix* sp. III were collected. See Table 1 for further details about locations. Circles represent the *16S* haplotype diversity within each location and their size is proportional to the number of individuals per site. Partitions inside the circles represent the relative proportion of each haplotype within each location. The dashed-grey line represents the Almeria-Oran Front (AOF). See Fig. 2 for details about haplotypes for each species. (**B**) MDS representation of the Ф_ST_ values at each location for each of the three species. Figures were created with the free software QGIS (http://qgis.osgeo.org/es/site/), and edited in Adobe Illustrator CS5.1 (http://www.adobe.com) for this study.

**Figure 2:**
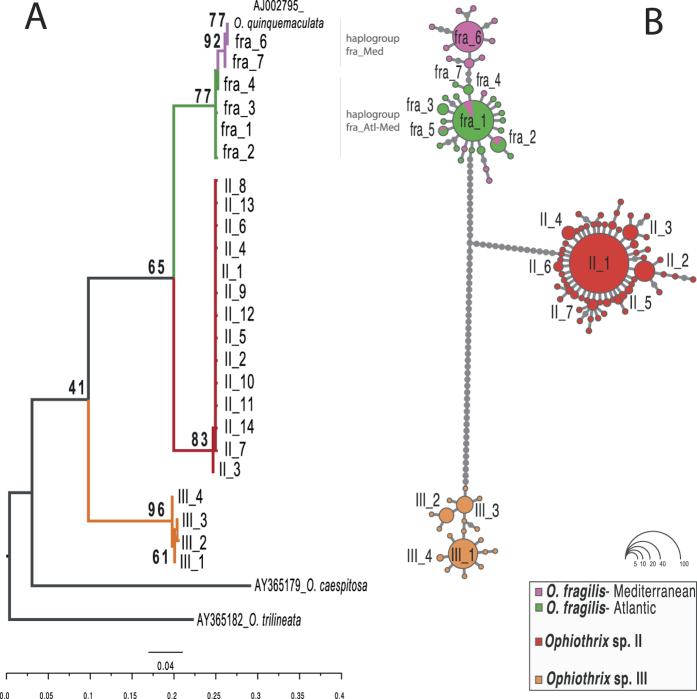
(**A**) Maximum Likelihood phylogenetic tree of the most frequent haplotypes of three species of *Ophiothrix* resulting from the *16S* marker. Only bootstrap support values >40 are indicated in the main nodes. (**B**) Haplotype network for the three *Ophiothrix* species based on the complete *16S* alignment. Circles are proportional to the number of individuals per haplotype. Grey dots correspond to mutational steps.

A total of 310 bp were obtained for the *16S* fragment for the three species of *Ophiothrix* considered here. For the *COI* marker only three sequences (797 bp) of *Ophiothrix* sp. III from the Cantabrian Sea (two from station DEM91 and one from DEM45; see Table 1 and Fig. 1) were obtained, which were added to a previous dataset of 148 sequences of *O. fragilis* and *Ophiothrix* sp. II by Pérez-Portela *et al.*^15^. ML phylogenetic trees based on both the most common haplotypes of *16S* and the total number of haplotypes of the *16S* and *COI* showed three moderately supported clades, namely *O. fragilis*, *Ophiothrix* sp. II, and *Ophiothrix* sp. III. Although the three species can unambiguously be considered as different species due to the genetic distance between the three mitochondrial lineages, morphology and bathymetric segregation (see Results below), their relative phylogenetic relationships within the genus could not be resolved due to the low support of the nodes (Fig. 2A, supplementary Figs S2 and S3). Two different haplogroups were recovered for the *16S* in *O. fragilis* corresponding to samples from the Mediterranean and from a mixture of samples from the Atlantic and Mediterranean (Fig. 2; fra_Med and fra_Atl-Med); these haplogroups, though, were not recovered in the *COI* phylogenetic tree because samples of this species from the Mediterranean were not available. Difficulties to obtain good quality sequences from additional genes, such as the *COI* from Mediterranean samples of *O. fragilis*, and the nuclear Histone 3, Internal Transcribed Spacers 1 and 2, prevented to generate better supported phylogenies to clarify the evolutionary relationships among lineages.

PTP analyses based on both the *16S* and *COI* markers corroborated the existence of three species corresponding to the three main clades identified in the phylogenetic trees (supplementary Table S1, Figs S4 and S5). Inter-specific genetic distances (K2p) between the three species ranged from 11–20% for the *16S,* and from 19–22% for the *COI* (Table 2); these values were lower than those between these three species and other congeneric species (see supplementary Table S2 for a complete list of divergence values of the *COI*). Intra-specific values (K2p) were ca. ten fold lower than inter-specific ones and ranged from 0.9–1.6% for *16S,* and between 0.11–0.17% for *COI* (Table 2).

### Genetic diversity and population connectivity

A total of 237 new individuals were sequenced for the *16S*, corresponding to 137 from *O. fragilis* (34 haplotypes), 80 from *Ophiothrix* sp. III (19 haplotypes), and 20 from *Ophiothrix* sp. II, the latter collected at FER; this dataset was analysed together with the 215 individuals of *Ophiothrix* sp. II sequenced by Pérez-Portela *et al.*^15^ (Table 1). The three different species did not occur in the same locality except for FER, where both *O. fragilis* (16 individuals) and *Ophiothrix* sp. II (33 individuals) were found in sympatry. Out of the total 111 haplotypes found for all species, 85 (76.6%) were private. Values of genetic diversity are detailed in Table 1.

The haplotype network based on the joined *16S* alignment of the three species was structured in three different main clades (species) lacking intermediate haplotypes (Fig. 2B). Divergence among clades was caused by 27 mutations between *O. fragilis* and *Ophiothrix* sp. II, and 51 between *Ophiothrix* sp. III and the other two species (Fig. 2B). The *O. fragilis* network exhibited two star-like patterns (haplogroups: fra_Md and fra_Atl-Med) connected by two mutational steps. In both cases, two dominant haplotypes were located at the center of the haplogroups: fra_6, appearing only at Mediterranean locations; and fra_1, broadly distributed across the Atlantic and Mediterranean basins, being the most common haplotype in the Atlantic basin. Apart from fra_1 (present in the Atlantic and in the Mediterranean populations of PSE and PLA), only two other haplotypes were shared between the two basins: fra_2, in three Atlantic populations (FER, COR, and ROS) and the Mediterranean PSE; and fra_5, which appeared in the Atlantic KRI and the Mediterranean PLA (Figs 1A and 2B). *Ophiothrix* sp. II showed a clear star-like pattern with II_1 as the ancestral haplotype, present in a similar proportion in all populations in both basins, surrounded by low-frequency (II_2 to II_7) and private haplotypes (Figs 1A and 2B). *Ophiothrix* sp. III revealed a network with a few dominant haplotypes (III_1 to III_4) surrounded by private haplotypes (Fig. 2B). Similar contributions of III_1, III_2 and III_3, the most common haplotypes in *Ophiothrix* sp. III, were observed in all populations (Fig. 1A).

AMOVA results showed no significant difference in the genetic structure between the Atlantic and Mediterranean basins and among sites for *Ophiothrix* sp. II and *Ophiothrix* sp. III, which retained 100% of their genetic variability within sites (Table 3). In contrast, *O. fragilis* showed significant differences between the two basins (58% of total genetic variation), and within sites (41%) (Table 3).

Ф_ST_ values confirmed AMOVA results, showing only significant differences between pairwise comparisons among populations of *O. fragilis* corresponding to different basins (Fig. 1B; Table 4, supplementary Tables S3 and S4). These results indicated panmixia across the geographical study area in *Ophiothrix* sp. II and III, while a strong phylogeographic break was observed between basins in *O. fragilis* (Fig. 1B). Genetic differentiation was weak although significantly correlated to geographical distances when the complete *O. fragilis* dataset was considered (Mantel test: *r* = 0.0004; p = 0.043), but this pattern of isolation by distance was not significant when basins were analysed separately (Atlantic: *r* = 0.0000; p = 0.588; and Mediterranean: *r* = −0.0002, p = 0.547).

**Figure 3:**
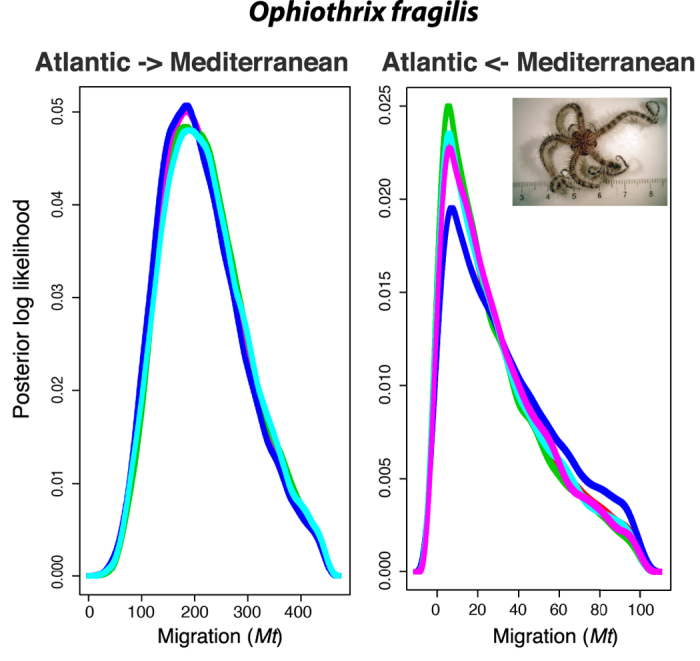
Results of migration analyses between the Atlantic and Mediterranean basins in *Ophiothrix fragilis* based on *16S* sequences. The two graphs represented the final results of LAMARC for 5 different replicates (left graph: Migration from the Atlantic basin to the Mediterranean basin; and right graph: Migration from the Mediterranean basin to the Atlantic basin).

LAMARC results for *O. fragilis* showed an almost unidirectional migration for mitochondrial DNA from the Atlantic Ocean to the Mediterranean Sea according to the posterior likelihood values obtained (Fig. 3).

### Demography

**Figure 4:**
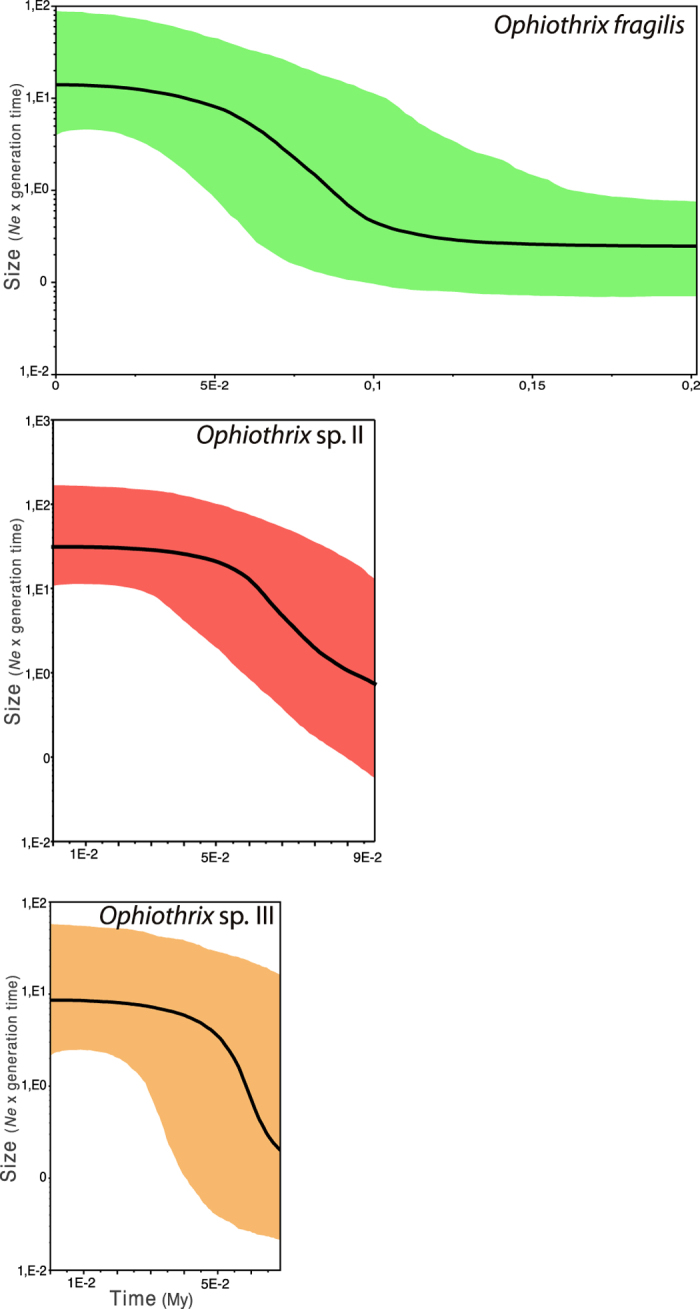
Demographic analyses of the three *Ophiothrix* species based on Bayesian Skyline plots of the *16S* marker. Time is measured in million years (Mya). Black lines illustrate mean size estimations, and colour shadows show 95% confidence interval.

The neutrality Rozas’ R^2^ test was significant for the three *Ophiothrix* species, which can be interpreted as an evidence of past demographic expansion (see *16S* results in Table 1). The mismatch distribution based also on the *16S* marker showed a unimodal distribution in *Ophiothrix* sp. II and III, also attributable to demographic expansion (supplementary Fig. S6). In the case of *O. fragilis*, though, despite showing a clear bimodal distribution when all populations were analysed together, a unimodal distribution, likely due to a past demographic expansion, was detected for Atlantic samples separately analysed, a pattern not observed for the Mediterranean dataset (supplementary Fig. S6). BSP results further confirmed demographic expansion in the three species. According to our analyses based on the *16S* fragment, demographic expansion for the three species happened more than 50,000 years ago, remaining stable for approximately the last 40,000 years (Fig. 4). These analyses also suggest differences in female effective population sizes among species, *Ophiothrix* sp. II displaying the largest effective population size (Fig. 4). BSP results based on the *COI* of *Ophiothrix* sp. II also detected a demographic expansion but much earlier, ca. 100,000–180,000 years ago (see supplementary Fig. S7), than for the *16S* marker. These differences may be related with the fact that there is not a well-calibrated molecular clock for these markers in ophiuroids, and the values obtained need to be taken as an approximation. For *O. fragilis*, estimations based on *COI* could not be performed due to the absence of coalescence in the dataset.

## Discussion

Our study revealed unexpected biodiversity in *Ophiothrix* along the Atlantic-Mediterranean area, with the occurrence of three different lineages that may be considered as species. Despite the closely evolutionary relationships among them, they displayed different geographic and bathymetric distributions, and contrasted phylogeographic patterns for the mitochondrial gene *16S*, a fact that should be contrasted in the future with the analysis of nuclear markers to infer if they are congruent with mitochondrial data. Our study represents an important contribution in the context of phylogeography of ophiuroids, since this has only been addressed for a few taxa worldwide^15^,^23^,^24^,^25^,^26^,^27^,^28^,^29^,^30^.

Two of the species found in this study, *O. fragilis* and *Ophiothrix* sp. II, were distinguished in great detail in a previous study^15^, while *Ophiothrix* sp. III is reported here for the first time. Phylogenetic inference using *16S* and *COI* combined with species delimitation analyses unambiguously confirmed that these three organisms are in fact three closely related congeneric species (Fig. 2, supplementary Figs S2–S5). In addition, no haplotype was shared among them and levels of inter-specific genetic divergence in *16S* and *COI* were high and larger that those measured at the intra-specific level. In this sense, divergence between *Ophiothrix* in our study was an order of magnitude greater (19–22% for *COI*) than the limit commonly accepted to discriminate species in echinoderms (0.9–1% for *COI*^31^). Importantly, remarkable morphological and ecological (bathymetric distribution) differences supporting the genetic divergence exist (supplementary Fig. S1; see Methods section), although they were not fully discussed in this work. Forthcoming and on-going morphological studies will lead to the formal description of both *Ophiothrix* sp. II and *Ophiothrix* sp. III (Manjón E. *et al.* in preparation).

The three *Ophiothrix* species studied here overlapped at least partially across their geographical distribution range, although they were in general bathymetrically segregated (Fig. 1A; Table 1). *Ophiothrix* sp. II is an intertidal and shallow subtidal species (0–60 m)^15^, whereas *O. fragilis* commonly occurs from the intertidal to the deep subtidal in the NE Atlantic, and from ~60 m to 100–130 m depth in the NW Mediterranean, being absent from shallower waters in the Mediterranean area. Such a bathymetric segregation between closely related ophiuroids is not the first time to be noticed. Muths *et al.*^27^ also reported the occurrence of two brittle stars of the genus *Acrocnida* that appeared in the intertidal (*Acrocnida spatulispina*) and the subtidal (*Acrocnida brachiata*) along the English Channel and the coast of Brittany, that had little overlapping in their distribution. Similarly, the widely distributed Atlantic-Mediterranean *O. fragilis* and *Ophiothrix* sp. II, only occurred in sympatry in the intertidal and shallow subtidal of the Atlantic coast of Ferrol and at ~50–60 m in the NW Mediterranean. Interestingly, preliminary results of *in vitro* cross-fertilization experiments indicated fertilization success in both intra- and inter-specific trials (the latter with less success) using a higher concentration of sperm than in natural conditions (authors’ unpublished data), which may suggest that these species are able to hybridize despite their relatively high genetic divergence (11% for *16S,* 19% for *COI*; Table 2). These preliminary results are in agreement with the ones reported for the above-mentioned *Acrocnida* ophiuroids, where it was proved that hybrids occurred despite the two species showing a divergence of about 20% for *COI*^27^,^28^. Prezygotic barriers, such as reproductive asynchrony and/or ecological preferences (*i.e.* bathymetric segregation), were suggested to be limiting hybridization in *Acrocnida*^28^; these barriers might also be preventing the opportunity to hybridize between *O. fragilis* and *Ophiothrix* sp. II, since the principal annual recruitments for these species do not overlap^22^,^23^ and their habitat preferences differ. Prezygotic barriers have been widely documented in other echinoderms, especially for echinoids, including examples on habitat separation between species but also on gametic isolation due to the evolution of molecules involved in gamete recognition *e.g.* refs 32 and 33.

A possible explanation for the contrasting distribution in *O. fragilis* and *Ophiothrix* sp. II was already anticipated by Pérez-Portela *et al.*^15^, who suggested that their speciation processes may have taken place during the Mio-Pliocene transition, a period coincident with the isolation of the North Atlantic area into two main basins. Briefly, after the opening of the English Channel and the subsequent contact of the two North Atlantic basins previously isolated, *Ophiothrix* sp. II, originally preadapted to the warmer and shallower conditions of the southern basin of the North Atlantic, colonized the shallow intertidal-subtidal across the South European coast and the Mediterranean Sea after the opening of Gibraltar Strait following the Messinian Salinity Crisis. On the other hand, *O. fragilis*, preadapted to colder temperatures and deeper waters from the northern basin of the North Atlantic, subsequently colonized the deeper Mediterranean subtidal, where conditions are more similar in temperature to those currently found in the shallow Atlantic subtidal^15^. Thus, the distribution of these two *Ophiothrix* spp. found in our study confirms the hypothesis already presented by Pérez-Portela *et al.*^15^. Interestingly, a similar hypothesis was suggested to explain the current ecological segregation observed for the two *Acrocnida* species mentioned earlier^28^.

*Ophiothrix* sp. III was exclusively found in deep subital waters (>100 m) of the Atlantic Cantabrian Sea and Portugal shore, as well as also in the Mediterranean Alboran Sea, which lies just behind the AOF, typically considered as one of the most important biogeographic barriers separating the Atlantic from Mediterranean basins^1^. Hence, our observations for *Ophiothrix* sp. III do not allow us venturing any hypothesis about the current distribution of this species within the Mediterranean, although its absence in NW Mediterranean deep subtidal areas, where *O. fragilis* is commonly found, might indicate that its distribution limit in the Mediterranean is at the AOF, as observed in other marine invertebrates *e.g.* refs 9, 13 and 34. Nevertheless, further sampling in deep waters of the SW Mediterranean and Eastern Mediterranean sub-basin would be necessary to have a complete picture of the geographical distribution of this species.

Overall, the three *Ophiothrix* species showed high levels of genetic diversity comparable to those reported in other brittle stars *e.g.* refs 24 and 29, although they showed different phylogeographic patterns. Panmixia in the shallow-water *Ophiothrix* sp. II, with no barrier preventing gene flow between Atlantic and Mediterranean populations^15^, contrasts with the genetic structure between basins observed in *O. fragilis*, supported by the significant values of AMOVA and Ф_ST_ (Fig. 1B; Table 3 and supplementary Tables S3 and S4). Examples of marine species displaying similar dispersal capabilities but contrasted patterns of genetic structure are widely documented in the Atlantic-Mediterranean area (see Patarnello *et al.*^1^ for a review). Among these, the studies on the broadcast spawning invertebrates *Nephrops norvegicus* (Norwegian lobster) and the *Homarus gammarus* (European lobster) showed no clear differentiation pattern between basins in the former while well differentiated groups occurring in the different basins were observed for the latter^12^,^20^. More recently, Fernández *et al.*^8^ described admixture with high gene flow for *Chiton olivaceous,* and a ‘chaotic patchiness’ pattern with high genetic variability and private haplotypes in all sites for *Lepidopleurus cajetanus*, two closely related chiton molluscs occurring in sympatry with presumably limited dispersal ability due to their lecithotrophic larva.

Large population size, high dispersal potential due to a long-lasting larval phase and shallow-water near shore currents along the shore, and a past demographic expansion event were used as possible reasons to explain the genetic homogeneity observed in *Ophiothrix* sp. II^15^. Effective population size estimations using the BSP confirmed the existence of larger population size in *Ophiothrix* sp. II when compared to the other two species (Fig. 4). *Ophiothrix fragilis* with smaller effective population size might be more vulnerable to the effect of genetic drift, a process that changes allele frequencies promoting genetic divergence between populations over short periods of time^35^.

Populations showing panmixia, as in *Ophiothrix* sp. II, have been reported for many echinoderms with planktotrophic larvae. In the holothurian *Holothuria mammata* genetically homogeneous populations were reported along the Macaronesian Islands, Algarve, and Western Mediterranean, although a significant break in genetic structure was detected in the Aegean Sea^16^. In other latitudes, the congeneric brittle star *Ophiothrix suensonii* also displayed lack of genetic differentiation across Florida and the Caribbean up to 1,700 km apart, combined with significant differences in genetic structure for some populations^29^.

*Ophiothrix fragilis* likely displayed genetic homogeneity within basins due to a long-lasting planktotrophic larva that promotes connectivity between distant populations, but clear divergence between basins. Examples of divergence between basins are not scarce in the literature, and have been reported for a diversity of marine invertebrates *e.g.* refs 2, 4, 11, 13 and 36. Two of the most common sea urchins across the Atlantic-Mediterranean area, *Paracentrotus lividus* and *Arbacia lixula*, showed no genetic differentiation over distances of thousands of km but divergence between basins despite having presumable high dispersal ability due to a long-lasting planktotrophic larva, demonstrating the strong disruptive effect of the Gibraltar Strait and/or Alboran Front^3^,^6^,^11^. The sea star *Astropecten auranciacus* also showed some significant differentiation of Atlantic versus Mediterranean populations and Western versus Eastern Mediterranean sub-basins that could not be attributed to any of the specific marine barriers targeted in their study (Strait of Gibraltar, AOF, Siculo-Tunisian Strait), but instead of isolation by geographical distance^5^. In contrast, divergence in *O. fragilis* did not follow a strong pattern by isolation by distance. The weak correlation detected with the Mantel test (*r* = 0.0004, *p* = 0.043) for the whole species dataset was magnified by the fact that all Mediterranean populations (very divergent from the Atlantic ones) were geographically close to each other, which may explain why this pattern of isolation by distance was not maintained within basins. Hence, we hypothesize the disruptive effect of the Gibraltar Strait and/or the AOF to be the most plausible genetic barrier explaining the divergence detected between basins in *O. fragilis*.

Nevertheless, the pattern observed for *O. fragilis* is complex and cannot be explained by a simple process of divergence between basins due to current genetic isolation. The two main haplogroups identified represent phylogeographical discontinuities that typically evolved in response to long-term extrinsic barriers to gene flow, although the present day structure may be affected by a redistribution of genetic diversity by contemporary contact. The asymmetrical distribution of haplogroups is congruent with a process of past vicariance due to allopatry between basins followed by secondary contact within the Mediterranean^37^, which is supported by the bimodal mismatch distribution (supplementary Fig. S6). This pattern in *O. fragilis* resembles that in *Marthasterias glacialis*, a widely distributed sea star in the Atlantic-Mediterranean region, with two distant lineages occurring in the Mediterranean Sea, but only one of them endemic from the Mediterranean^14^. Lineage splitting in *M. glacialis* was attributed to vicariance and secondary contact during Pleistocene glaciations (800–12 Kya) that caused sea level drops off up to 150 m with dramatic range shifts along the European coastline, ultimately interrupting circulation of species across the most important marine European corridors^38^. Hence, most of the present-day genetic patterns of marine coastal populations across the Atlantic-Mediterranean arch are the result of periodical limitations of larval interchange and migration across the Gibraltar Strait^1^,^10^. Our results for *O. fragilis* suggest a similar pattern to that observed in *M. glacialis*, with a recent demographic expansion during the last 50,000 years (Fig. 4), which could have followed a subsequent geographical expansion from the Atlantic to the Mediterranean. This is supported by the higher haplotype and nucleotide diversity in the Mediterranean (Table 1) due to the admixture of haplotypes from the two haplogroups in this basin, and by the unidirectional gene flow (migration) from the Atlantic to the Mediterranean detected in LAMARC (Fig. 3). However, the lack of a specific molecular clock for *16S* and *COI* in ophiuroids make that estimations of demographic expansions need to be taken with caution and can be only used for comparison among species. At present, the predominant gene flow from the Atlantic to the Mediterranean might be maintained by major circulation currents across the Gibraltar Strait, due to a strong surface inflow from the Atlantic to the Mediterranean and water stratification^39^. The reverse current slides down 400–800 m depth, environmental conditions under which larvae of some echinoderms cannot survive^40^, might prevent the endemic Mediterranean haplogroup to disperse further in the Atlantic. Despite the disruptive effect of marine circulation across the Gibraltar Strait, the bathymetric fractioning between Atlantic (intertidal and shallow subtidal down to 60 m) and Mediterranean populations (deep subtidal, >60 m) in *O. fragilis* might be reinforcing the genetic divergence observed between basins by limiting the connectivity among populations^41^. The absence of the Mediterranean haplogroup (fra_Med) in Atlantic populations could be also related to the adaptation of lineages to different environmental conditions between habitats (shallow vs deep water) or basins, a hypothesis that cannot be completely ruled out. For instance, in the European anchovy water temperature shapes the contemporary distribution of mitochondrial DNA lineage frequencies due to strong selection on some codons^42^.

In conclusion, we present here important insights in *Ophiothrix* diversity, geographic and bathymetric distribution, and most relevant processes shaping intraspecific diversity of three Atlantic-Mediterranean species. It is a remarkable example of how closely related species, with similar biological features, display divergent phylogeographic patterns inferred from the mitochondrial markers due to the different effect of both historical and/or contemporary events. Future studies should be directed to determine the distribution of *O. fragilis* and the new species *Ophiothrix* sp. III across the whole Mediterranean basin by surveying under-sampled areas, with an especial emphasis in the deep subtidal. Also, efforts should be directed to investigate the genetic patterns across transition areas and biogeographic barriers such as the AOF, as well as the influence of other oceanographic fronts across the Mediterranean^21^. Importantly, further studies should develop and target nuclear markers for these three species since gene genealogies obtained for the mitochondrial markers, *16S* and *COI*, can be different from the complete history of species. Additional nuclear markers could refine connectivity estimates, as well as the potential for hybridization between species, mitochondrial introgression and effective population size^43^,^44^.

## Methods

### Sample collection

Samples of *Ophiothrix fragilis* were collected between 2005 and 2012 from eight shallow and deep sites (Fig. 1A and Table 1), including four Atlantic locations [Kristineberg (KRI), Roscoff (ROS), Ferrol (FER), and Vigo collected on board the *R/V Cornide de Saavedra* (COR)], and four Mediterranean ones [Blanes (ABL), Barcelona-*INDEMARES* project (IMA), Planassa area (PLA), and Port de la Selva (PSE)]. KRI, ROS, and FER samples were collected by scuba-diving (8–10 m depth) and by hand in the intertidal; the rest of samples were collected by trawling on the *R/V Cornide de Saavedra* and on fishing trawlers (30 to 139 m depth). Samples of a species morphologically similar to *O. fragilis* and *Ophiothrix* sp. II (see Results section), were collected in 2013 from five deep subtidal sites (100–310 m depth; Table 1), four of them located in the Atlantic basin [by trawling during the *DEMERSALES* cruise (DEM45, DEM91, and DEM131) on board the *R/V Miguel Oliver*- *ERDEM3* project, and the ones from Portugal (POR) by a fishing trawler], while the Mediterranean population (PINDAL) was collected during the *INDEXARES* cruise on board the ship *Isla de Alborán* (Fig. 1A; Table 1). Complete specimens were preserved in absolute ethanol, and stored at −20 °C until processed.

Samples of *Ophiothrix* sp. II analysed here for comparative purposes correspond to the collection of Pérez-Portela *et al.*^15^ plus 20 extra individuals from FER (Table 1). These samples were collected by scuba-diving or in the intertidal from 11 different shallow-water locations (Fig. 1A; Table 1), including three Atlantic locations [Ferrol (FER), Cascais (CAS), and Armação de Pera (APA)] and eight Mediterranean ones [Ceuta (CEB), La Herradura (LH), Xábea (XB), Roses (RS), Cadaqués (CAD), Alcudia (AL), Ladiko (LK), and Kalytea Bay (KB)].

### Morphology and Biology

Specimens of *Ophiothrix* spp. used for DNA sequencing were morphologically inspected, although we do not provide here detailed morphological descriptions. Specimens of *O. fragilis* displayed typical characters of the species: rounded disc with diameter between 7–10.6 mm, wide and naked radial shields covering approximately half of the aboral disc area. They also had the typical slender spines in the central area of the aboral side of the disc and in the inter-radial fields, and pale colours from brown to whitish^15^ (supplementary Fig. S1 and Table S1). Specimens of *Ophiothrix* sp. II showed strikingly colour plasticity and were within the range of size described by Pérez-Portela *et al.*^15^, with disc diameter between 6.5–10 mm, small radial shields on the aboral side covering 1/3–1/4 of its surface, and partially covered by small spines and tubercles. The central and inter-radial fields of the aboral side was homogeneously covered by small spines. Specimens of *Ophiothrix* sp. III were in general larger than the other two species displaying a rounded disc reaching up to 18 mm in diameter, although disc diameter of specimens largely varied among sites. Their wide radial shields covered approximately half of the aboral side of the disc as in *O. fragilis*, but they presented tubercles and small spines on the shields as in *Ophiothrix* sp. II (supplementary Fig. S1 and Table S1).

The only information about the reproductive patterns of *O. fragilis* comes from studies on Atlantic individuals, and concluded that the reproductive dynamics of *O. fragilis* has four annual recruitments, the principal of which taking place between September-October, with a planktotrophic larvae with a moderately long lifespan often recruiting directly onto the discs and arms of large adults^45^. In fact, several young recruits were found on the disc of adult specimens of *O. fragilis* collected in Kristineberg (KRI) during morphological analysis of the samples. In contrast to that reported for *O. fragilis*, *Ophiothrix* sp. II has a main annual reproductive period (late spring-early summer), through a planktotrophic larva and with juveniles settling on top of sponges and adults living in small numbers between algae and under rocks^46^.

### DNA sequencing

Total DNA was extracted from tube feet using the REDExtract-N-Amp kit (www.sigma.com) following the manufacturer’s protocol. Fragments of the *16S* were amplified and sequenced using the primers designed by Pérez-Portela *et al.*^15^ for all new samples (Table 1). In addition, the mitochondrial *COI* was sequenced for species delimitation purposes for a subset of samples of *Ophiothrix* sp. III. Fragments of the *COI* were amplified and sequenced using the primers polyLCO/polyHCO^46^. PCR amplification reactions were performed in a 20-μL total reaction with 10 μL of REDEXtract-N-ampl PCR reaction mix, 0.8 μL of each primer (10 μM), 7.4 μL of ultrapure water, and 1 μL of DNA template. PCR temperature profile was 95 °C/5 min-(94 °C/1 min–46 °C/30 s–72 °C/2 min)*37 cycles–72 °C/8 min for *16S* and 94 °C/5 min-(94 °C/1 min–55 °C/1 min–72 °C/1 min)*35 cycles–72 °C/5 min for *COI*. PCR products were purified and sequenced by Macrogen, Inc. (Seoul, Korea) with the same primers used for amplification. New sequences have been deposited in GenBank (accession numbers KX577803–KX578016).

### Phylogeny and species delimitation

Due to the presence of individuals with different morphology, we first applied phylogenetic and species delimitation approaches to avoid mixing different species for further analyses.

Sequences were edited using *Geneious* vs. R8 and aligned with the Q-INS-I option of MAFFT. To detect different evolutionary units, *16S* and *COI* sequences were collapsed into haplotypes using *DnaSP* vs. 5.10.1^47^, and used for phylogenetic analyses, including as outgroups sequences of congeneric *Ophiothrix* species and other phylogenetically close ophiuroids. Outgroups varied between markers depending on the sequences available from Genbank (Fig. 2, and supplementary Figs 2S and 3S). The most appropriate evolutionary model for *16S* (HKY + I) and *COI* (GTR + I + G) were inferred using jModelTest^48^ via the Akaike Information Criterion (AIC). Maximum Likelihood (ML) phylogeny trees were estimated separately for *16S* and *COI* using PhyML as implemented in Seaview^49^, with 1,000 bootstrap replicates, and an optimised tree searching (Best of NNI and SPR). The resulting trees were edited in *FigTree* v.1.4.2 (http://tree.bio.ed.ac.uk/software/figtree/). Phylogenetic reconstruction was applied for the whole dataset of haplotypes of the *16S* and *COI* alignments, but also for the most frequent haplotypes of the *16S*, to simplify the interpretation of the intra-specific data.

The Poisson Tree Processes (PTP) model was applied to infer putative species boundaries among samples. This method is a powerful tool developed to identify cryptic species and to solve taxonomic ambiguities^50^,^51^. Based on the rooted ML phylogenetic trees of *COI* and *16S* sequences the PTP model was implemented in the webserver (http://species.h-its.org)^52^ using 100,000 MCMC generations, thinning of 100, and a burning of 0.1. Levels of genetic divergence between and within species of *Ophiothrix* detected from PTP were calculated based on the uncorrected Kimura 2 parameters (K2p) model using *MEGA* vs. 5.05^53^, with deviation estimated after 1,000 bootstrap replicates. The K2p model was applied to compare with previous studies in echinoderms.

### Genetic structure

We only used *16S* alignments due to its better resolution for populations’ genetic structure than *COI* as observed in previous studies with *Ophiothrix* and other echinoderms^3^,^15^. An unrooted haplotype network for the different species was constructed based on a ML tree with the program *Haploviewer* (www.cibiv.at/~greg/haploviewer).

All *16S* sequences were used to calculate number of haplotypes (*H*), number of private haplotypes (*Hp*), haplotype diversity (*Hd*), and nucleotide diversity (*π*) with *DnaSP*.

Differences in the genetic structure of populations were assessed by computing pairwise Ф_ST_ statistics in *ARLEQUIN* v 3.5^54^ between sites within each species. The corresponding *p*-values were evaluated by 10,000 permutations, and adjusted by a false discovery rate method^55^. The matrixes of the Ф_ST_ values were plotted graphically in a Multidimensional Scaling analysis (MDS). The fit of genetic differentiation between sites in *O. fragilis* (the only species displaying divergence between sites; see Results section) to a pattern of isolation by geographical distance was tested in *ARLEQUIN*, using the Mantel test procedure with 16,000 permutations. Geographical distances were calculated as the minimum distance in Km between sites over the sea.

Differentiation between the Atlantic and Mediterranean basins was assessed separately for each species by conducting hierarchical analyses of molecular variance (AMOVA) using genetic distances. Their significance was tested running 16,000 permutations in *ARLEQUIN*. Different components of the genetic variance were quantified per each species as follows: between basins (Atlantic vs Mediterranean), among sites within basins, and within sites. Due to the existence of genetic divergence between basins in *O. fragilis* (see Results), a Bayesian analyses was performed to evaluate migration rates between basins in LAMARC v 2.1.9^56^. The best evolutionary method for *O. fragilis* as inferred with jModelTest 2 (JC) was implemented. Three initial runs were performed to estimate the most likely priors for our dataset. The first run was performed with default parameters, and two additional runs were computed with most accurate parameters. Once accurate priors were obtained, they were implemented in a final run, with variation values of migration between 0 and 500, from the Atlantic to the Mediterranean basin, and 0 and 150 on the reverse way. The final run was based on five different replicates with 40 initial chains of 5,000 MCMC each, burning period of 500, and 5 final chains of 40,000 MCMC each with a burning period of 1,000. Two simultaneous heating searches (1 and 1.5) were performed per replicate. To visualise whether the run was long enough reaching a plateaus of probability, and to confirm the existence of at least 200 independent simulations (Effective Sample Size-ESS) for each parameter, results were summarised in Tracer v 1.5 (http://beast.bio.ed.ac.uk/Tracer). Migration rates (*Mt*) were expressed as the number of migrants per generation *Mt* = *n/u*, where *n* is the immigration rate per generation, and *u* the substitution rate.

### Demography

To test departures from a constant population size we used different approaches for the *16S*. First we estimated neutrality Rozas’ *R*^2^ statistic^57^ with *DnaSP* per population. Then the history of effective population size was assessed by: a) the classical pairwise mismatch distribution following the model of Rogers & Harpending^58^ in *ARLEQUIN*, in which populations tend to display unimodal and smoother distributions while stationary populations commonly show multimodal distributions; and b) the coalescent-based Bayesian skyline plots (BSP) using BEAUti v 2 and BEAST v 2.1.2^59^. For BSP priors included the implementation of the substitution models defined by jModelTest (JC, K80, and HKY for *O. fragilis*, *Ophiothrix* sp. II, and *Ophiothrix* sp. III, respectively), a strict clock model, and the constant skyline model. As no molecular clock has been calibrated for ophiuroids, a mutation rate of 1.25% per nucleotide per million years was used for the *16S*, following other studies in echinoderms^60^. Between 50 and 500 million MCMC generations were run per species, sampled every 5,000^th^–50,000^th^ step, and a burnin of 1,000–10,000. As explained for LAMARC, we ensured that the runs were long enough and above 200 ESS by visualizing the result outputs in Tracer. Tracer was also used to generate the evolution of effective population size under the skyline plot model, expressed as *N*_e_*T (T* = generation time) over time. BSP analyses were additionally run for the *COI* sequences available for *O. fragilis* and *Ophiothrix* sp. II. It was not performed for *Ophiothrix* sp. III because only three sequences were available for the species. A mutation rate of 2.48% per nucleotide per million years was used for the COI, following other studies in echinoderms^61^. Between 50 million and one billion MCMC generations were run per species, sampled every 5,000^th^–100,000^th^ step, and a burnin of 1,000–100,000 with the corresponding substitution model (GTR).

## Additional Information

**How to cite this article**: Taboada, S. and Pérez-Portela, R. Contrasted phylogeographic patterns on mitochondrial DNA of shallow and deep brittle stars across the Atlantic-Mediterranean area. *Sci. Rep.* **6**, 32425; doi: 10.1038/srep32425 (2016).

## Supplementary Material

Supplementary Information

## Figures and Tables

**Table 1:** Summary of the information related to the different localities surveyed and genetic variability for the *16S* for the three different *Ophiothrix* species.

Population	Code	Depth (m)	Coordinates	Geographical area	*N*	*H*	*p*	*Hd*	*π*	*R*^*2*^
***Ophiothrix fragilis***										
Kristineberg^†^	KRI	Intertidal	58°14′27.32“N 11°25′55.14“E	NE Atlantic- North Sea	22	9	5	0.749 ± 0.094	0.00368 ± 0.0007	0.1467**
Roscoff	ROS	Intertidal	48°45′9.55“N 3°58′38.10“W	NE Atlantic- English Channel	19	6	3	0.468 ± 0.140	0.00204 ± 0.0007	0.1667*
Ferrol^†^	FER	8–10	43°27′25.27“N 8°20′9.07“W	NE Atlantic	16	7	4	0.775 ± 0.088	0.00391 ± 0.0008	0.1650**
Vigo-Cornide^†^	COR	30–60	42°13′3.81“N 8°49′41.24“W	NE Atlantic	22	7	4	0.481 ± 0.131	0.00206 ± 0.0007	0.1618**
**Atlantic basin**					**79**	**21**	**16**	**0.625 ± 0.063**	**0.00294 ± 0.0004**	0.1040*
Blanes^†^	ABL	90	41°40′2.19“N 2°52′30.44“E	NW Mediterranean	9	4	2	0.750 ± 0.139	0.00821 ± 0.0033	0.2036
Indemares^†^	IMA	123–139	41° 18,5752′ N 2°25,6324′ E	NW Mediterranean	5	2	1	0.400 ± 0.237	0.00388 ± 0.0023	0.3000
Planassa^†^	PLA	80–100	41°38′47.92“N 2°54′40.61“E	NW Mediterranean	21	12	8	0.824 ± 0.084	0.01235 ± 0.0022	0.1345
Port de la Selva^†^	PSE	80–100	42°21′59.49“N 3°13′26.37“E	NW Mediterranean	23	4	1	0.577 ± 0.090	0.00849 ± 0.0012	0.1341**
**Mediterranean basin**					**58**	**17**	**12**	**0.698 ± 0.065**	**0.00960 ± 0.0012**	0.1037*
**TOTAL**					**137**	**34**	**28**	**0.777 ± 0.029**	**0.00979 ± 0.0005**	0.0088*
***Ophiothrix*****sp.** **II**										
Ferrol^†^	FER	8–10	43°27′25.27“N 8°20′9.07“W	NE Atlantic	33	12	7	0.758 ± 0.122	0.00408 ± 0.0011	0.1112*
Cascais	CAS	Intertidal	38°42′25.77“N 9°29′15.41“W	NE Atlantic	18	11	5	0.810 ± 0.093	0.00409 ± 0.0008	0.0539**
Armação de Pera	APA	10–15	37°5′41.52“N 8°21′37.31“W	NE Atlantic	20	9	3	0.758 ± 0.101	0.00394 ± 0.0009	0.0541**
Ceuta	CEB	18–25	35°52′52.19“N 5°19′10.20“W	Gibraltar Strait	19	8	3	0.672 ± 0.119	0.00321 ± 0.0009	0.0857**
La Herradura	LH	15–18	36°22′4.36“N 5°12′51.68“W	SW Mediterranean	20	6	2	0.447 ± 0.137	0.00241 ± 0.0009	0.0921*
Xábea	XB	10–20	38°47′39.48“N 0°12′18.10“E	SW Mediterranean	20	7	3	0.521 ± 0.135	0.00219 ± 0.0007	0.0818**
Roses	RS	10–20	42°14′11.17“N 3°15′45.80“E	NW Mediterranean	14	4	1	0.494 ± 0.151	0.00172 ± 0.0006	0.1246*
Cadaqués	CAD	2–15	42°17′27.85“N 3°17′43.95“E	NW Mediterranean	19	9	5	0.772 ± 0.094	0.00407 ± 0.0009	0.0700**
Alcudia	AL	15–18	39°52′1.28“N 3°11′39.87“E	W Mediterranean-Balearic Is.	26	13	5	0.720 ± 0.099	0.00359 ± 0.0008	0.0412**
Ladiko	LK	2–10	36°19′22.22“N 28°13′18.81“E	E Mediterranean	16	9	5	0.817 ± 0.095	0.00459 ± 0.0013	0.0745**
Kalytea Bay	KB	2–5	35°9′58.03“N 24°24′38.20“E	E Mediterranean	9	6	3	0.833 ± 0.126	0.00418 ± 0.0012	0.1111**
**TOTAL**					**215**	**58**	**42**	**0.679 ± 0.0390**	**0.00345 ± 0.00031**	0.0117**
***Ophiothrix*****sp. III**										
Demersales-45^†^	DEM45	153	43°36′44.7“N 8°29′49.5“W	NE Atlantic- Cantabrian Sea	20	6	3	0.679 ± 0.101	0.0040 ± 0.0009	0.098*
Demersales-91^†^	DEM91	310	43°53′27.24“N 5°34′53.77“W	NE Atlantic- Cantabrian Sea	22	5	5	0.745 ± 0.093	0.0039 ± 0.0007	0.1486**
Demersales-131^†^	DEM131	131	43°30′57.0“N 2°53′32.4“W	NE Atlantic- Cantabrian Sea	23	8	5	0.744 ± 0.080	0.0039 ± 0.0006	0.1472**
Peniche^†^	POR	100–150	39°27′53“N 9°12′48.33“W	NE Atlantic	7	4	1	0.714 ± 0.181	0.0054 ± 0.0018	0.2417
PINDALBV17^†^	PINDAL	169	36°08′0“N 30°2′0”E	SW Mediterranean	8	4	1	0.750 ± 0.139	0.0036 ± 0.0008	0.2311**
**TOTAL**					**80**	**19**	**15**	**0.714 ± 0.047**	**0.00407 ± 0.0004**	0.100**

*N* number of individuals, *H* number of haplotypes, *p* private haplotypes, *Hd* haplotype diversity, *π* nucleotide diversity, *R*^*2*^ Ramos-Onsins & Rozas statistic.

For *Ophiothrix* sp. II and *Ophiothrix* sp. III information of the Atlantic and Mediterranean basins is not presented since these two species did not displayed significant differences between geographical areas. ^†^New samples sequenced in this study. ^*^p < 0.05 and ^**^p < 0.01.

**Table 2:** Genetic distances (±standard error) between *Ophiothrix* spp. based on a Kimura 2-Parameters model for *16S* and *COI* markers.

16S	*O. fragilis*	*Ophiothrix* sp. II	*Ophiothrix* sp. III	*O. trilineata*	*O. caespitosa*
*O. fragilis*	**(0.0158** **±** **0.003)**				
*Ophiothrix sp. II*	0.111 ± 0.018	**(0.0091** **±** **0.001)**			
*Ophiothrix sp. III*	0.195 ± 0.027	0.203 ± 0.029	**(0.0089** **±** **0.002)**		
*O. trilineata*	0.308 ± 0.036	0.324 ± 0.039	0.318 ± 0.038	—	
*O. caespitosa*	0.262 ± 0.031	0.338 ± 0.039	0.295 ± 0.035	0.385 ± 0.043	—
**COI**	***O. fragilis***	***Ophiothrix* sp. II**	***Ophiothrix* sp. III**	***O. trilineata***	***O. caespitosa***
*O. fragilis*	**(0.0175** **±** **0.003)**				
*Ophiothrix sp. II*	0.189 ± 0.022	**(0.0117** **±** **0.001)**			
*Ophiothrix sp. III*	0.202 ± 0.023	0.220 ± 0.024	**(0.0112** **±** **0.004)**		
*O. trilineata*	0.237 ± 0.026	0.280 ± 0.029	0.264 ± 0.027	—	
*O. caespitosa*	0.267 ± 0.029	0.269 ± 0.029	0.253 ± 0.026	0.293 ± 0.032	—

Inter-specific distance values are presented below the diagonal. Numbers along the diagonal in brackets represent intra-specific variation for the three species analysed in this study.

**Table 3:** Results of the AMOVA analyses for each of the three *Ophiothrix* species based on the *16S*.

Source of variation	d.f.	Sum of squares	Fixation index	% of variation	*p*-value
***Ophiothrix fragilis***					
Among basins	1	84.1	FCT = 0.580	58.02	0.028*
Among sites within basins	6	6.7	FSC = 0.015	0.65	0.293
Within sites	128	114.2	FST = 0.587	41.3	0.000**
***Ophiothrix*****sp**. **II**					
Among basins	1	0.512	FCT = −0.0007	0.000	0.483
Among sites within basins	9	4.854	FSC = −0.0007	0.000	0.487
Within sites	182	99.323	FST = −0.0013	100	0.535
***Ophiothrix*****sp**. **III**					
Among basins	1	0.315	FCT = −0.0158	0.000	1.000
Among sites within basins	3	0.933	FSC = −0.0295	0.000	0.944
Within sites	74	44.930	FST = −0.0458	100	0.966

**Table 4:** Ф_ST_ values between pairs of populations for *Ophiothrix fragilis* based on the *16S*.

*Ophiothrix fragilis*							
	ABL	PLA	IMA	PSE	ROS	FER	KRI
PLA	−0.02310	—					
IMA	−0.04994	0.01724	—				
PSE	0.01959	−0.01746	0.11168	—			
ROS	0.74571**	0.49423**	0.86336**	0.54393**	—		
FER	0.67420**	0.45448**	0.78977**	0.49536**	0.04975	—	
KRI	0.68246**	0.46144**	0.79129**	0.50192**	0.00596	0.04456	—
COR	0.75560**	0.50949**	0.86475**	0.55582**	−0.01535	0.05551	0.01485

^**^Significant values after applying the false discovery rate method.
