# Supplementary material for: Contrasted phylogeographic patterns on mitochondrial DNA of shallow and deep brittle stars across the Atlantic-Mediterranean area
Source: Sci Rep. 2016 Sep 2;6:32425. doi: 10.1038/srep32425 (PMC5009426; doi:10.1038/srep32425)
Supplement: Supplementary Information [file srep32425-s1.pdf]

Contrasted phylogeographic patterns on mitochondrial DNA of shallow and deep brittle stars across the Atlantic-Mediterranean area

Sergi Taboada, Rocío Pérez-Portela

Supplementary material

Figure S1. Pictures of three specimens of the three *Ophiothrix* spp. investigated in this study. **A.1** *Ophiothrix fragilis* (specimen from Roscoff, Atlantic Ocean). **B.1** *Ophiothrix* sp. II (specimen from Alcudia, Mediterranean Sea). **C.1** *Ophiothrix* sp. III (specimen from Demersales-91, Cantabrian Sea). **A.2**, **B.2** and **C.2** detail of the disc in *Ophiothrix fragilis*, *Ophiothrix* sp. II, and *Ophiothrix* sp. III, respectively.

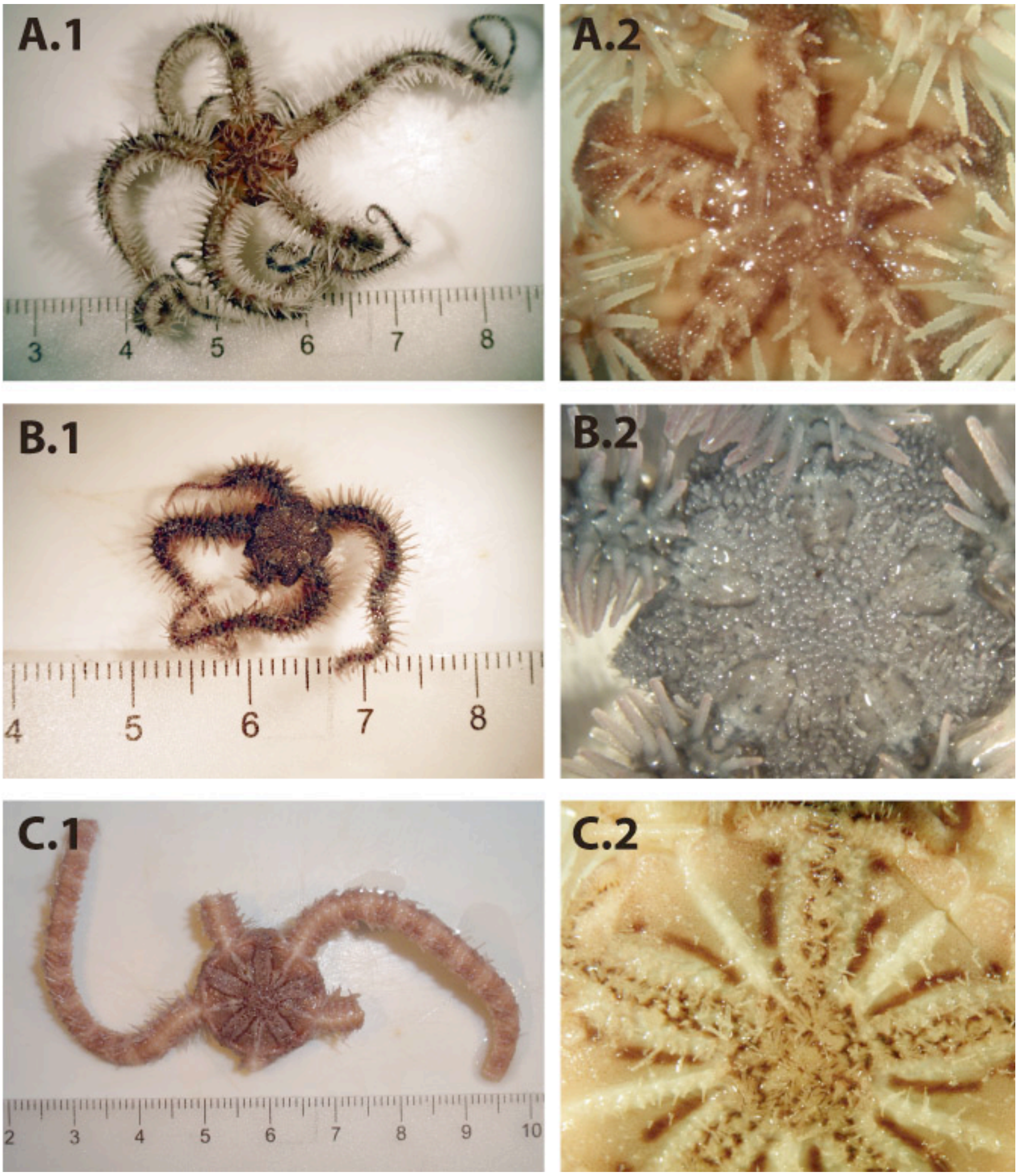

Figure S2. Maximum Likelihood phylogenetic tree of the genus *Ophiothrix*, including *Ophiothrix fragilis*, *Ophiothrix* sp. II, and *Ophiothrix* sp. III, based on *16S* sequences. Only bootstrap support values >30 are indicated in the main nodes. Sequences obtained from Genbank are presented with Accession numbers.

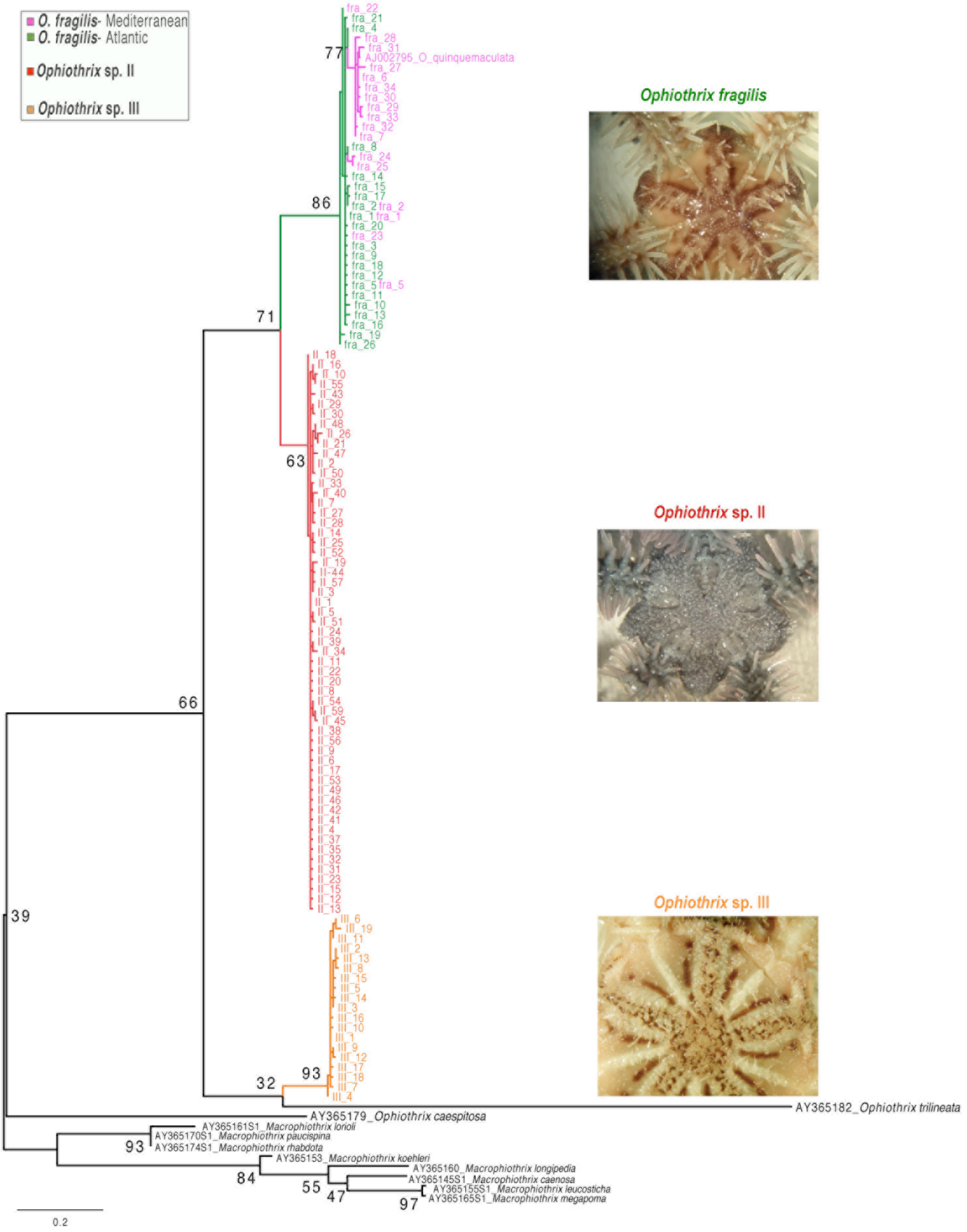

Figure S3. Maximum Likelihood phylogenetic tree of the genus *Ophiothrix*, including *Ophiothrix fragilis*, *Ophiothrix* sp. II, and *Ophiothrix* sp. III, based on *COI* sequences. Only bootstrap support values >30 are indicated in the main nodes. Sequences obtained from Genbank are presented with Accession numbers.* Representative sequences of *O. fragilis* obtained from Muths *et al*. (2009).

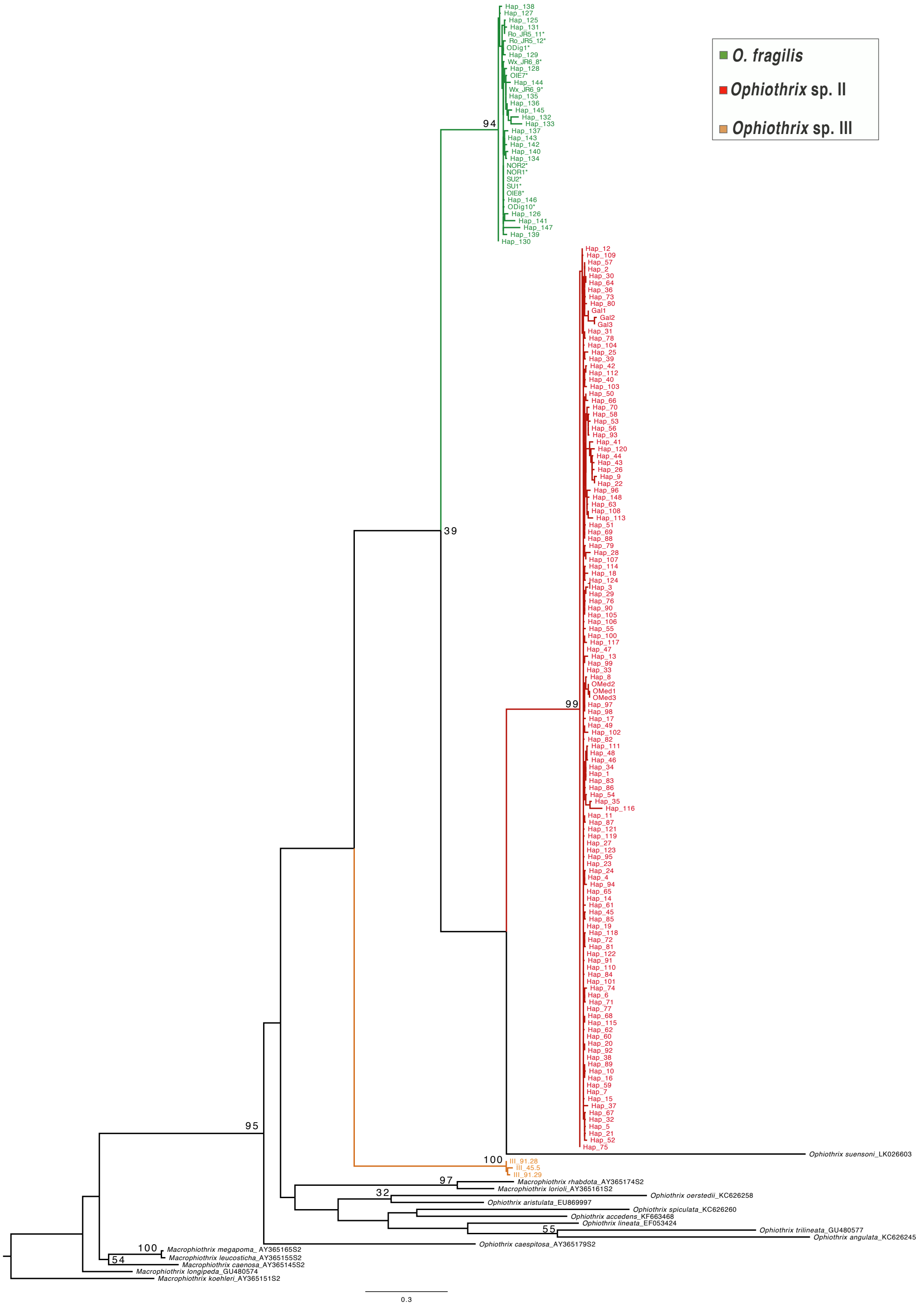

Figure S4. **Results of the PTP analyses based on the ML topology for the *16S*. Putative species clusters are indicated using transitions between black-coloured to red-coloured branches.**

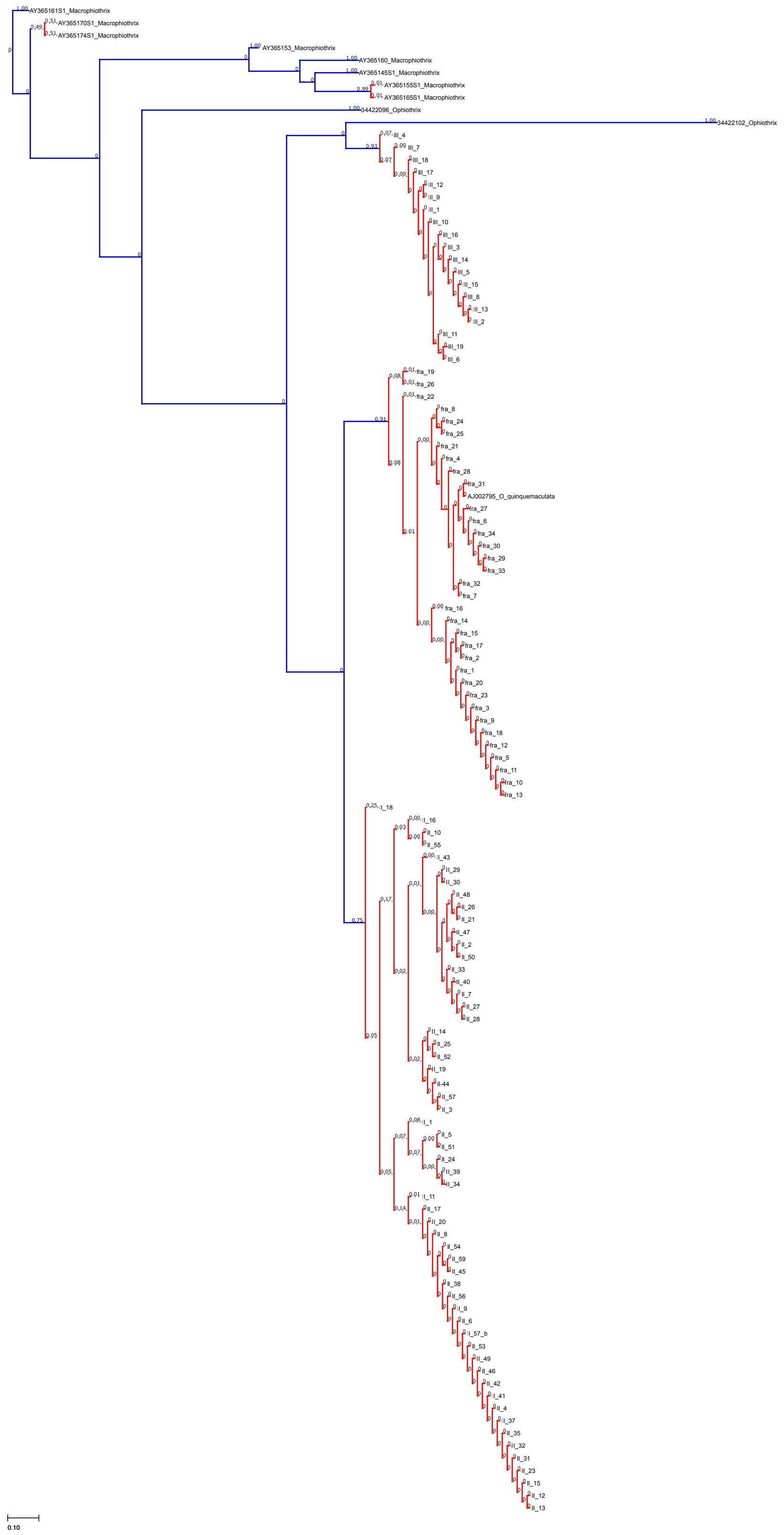

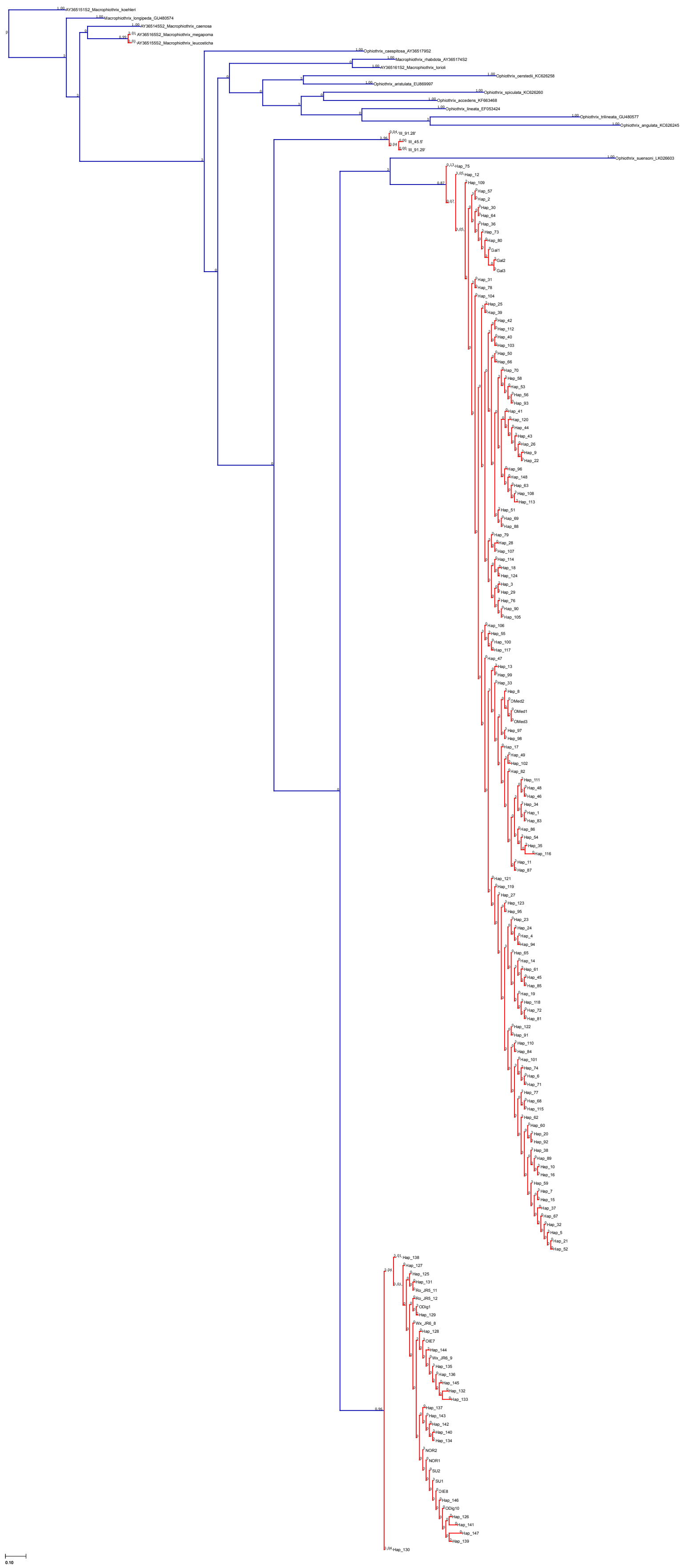

Figure S5. Results of the PTP analyses based on the ML topology for the *COI*. Putative species clusters are indicated using transitions between black-coloured to red-coloured branches.

Figure S6. Pairwise mismatch distributions for *Ophiothrix fragilis*, *Ophiothrix* sp. II, and *Ophiothrix* sp. III based on *16S* sequences. Observed data and theoretical expected distributions are represented by discontinuous and solid lines, respectively. For *Ophiothrix fragilis*, mismatch distributions for the Atlantic and Mediterranean basins are also represented separately.

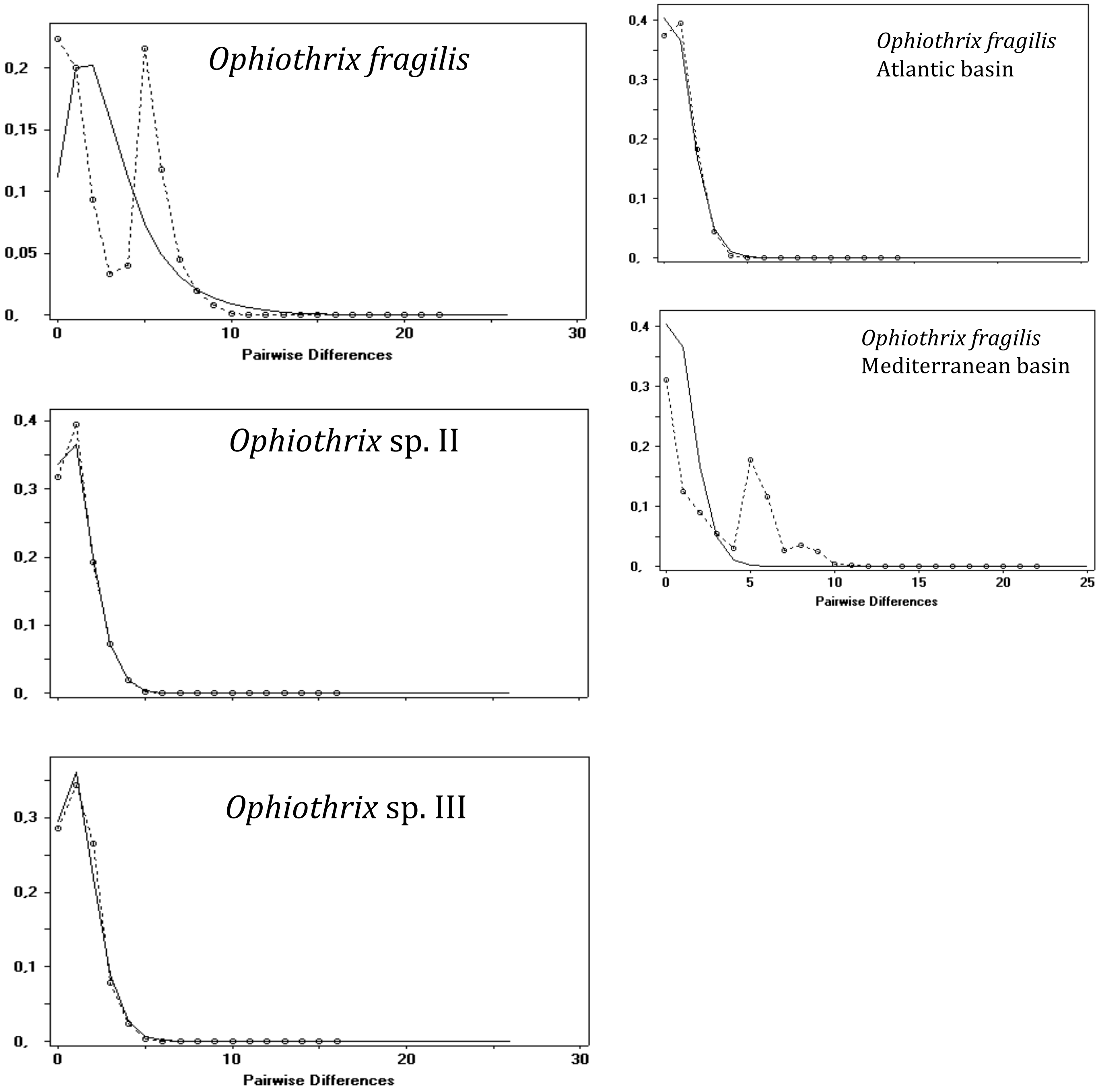

Figure S7. Demographic analyses of *Ophiothrix* sp. II based on Bayesian Skyline plot of the *COI* marker. Time is measured in million years. Black line illustrates mean size estimations, and blue lines show 95 % confidence interval.

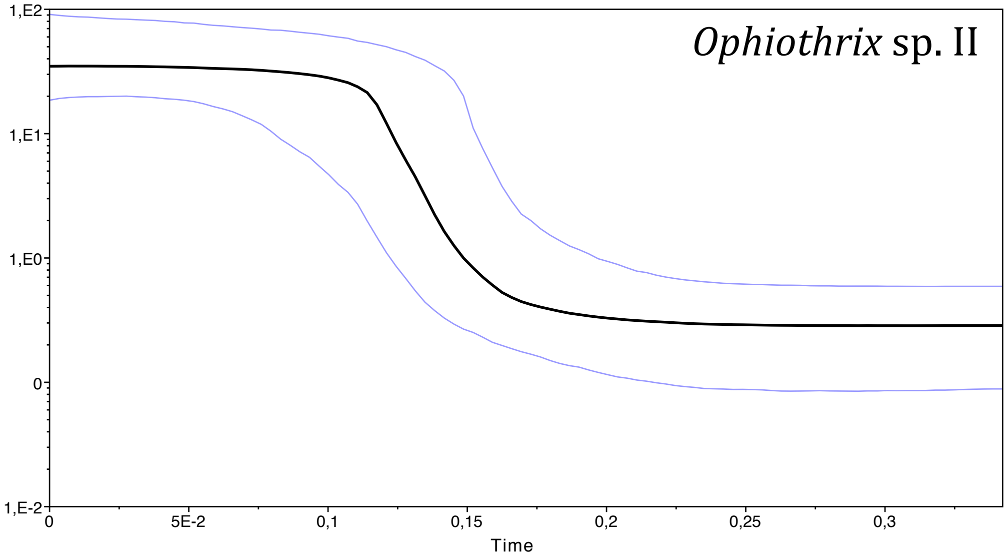

Table S1. Morphological and distribution differences among *Ophiothrix* species find in this study.

Lineage	Colour	Disc shape	Radial shields	Disc diameter	Disc spines	Known distribution
O. fragilis	Pink, pale pink, purple, beige, white and/ or whitish	Mostly rounded	Wide and naked	7.1 mm -10.6 mm	Large and elongated	Intertidal and shallow subtidal of North East Atlantic and North Sea, English Channel, and coast of Galicia (Atlantic coast of the Iberian Peninsula). Deep subtidal in the North Western Mediterranean (80-140m depth).
Ophiothrix sp. II	Large variation in colours and patterns: grey, dark brown, bright brown, blue, dark blue, bright green, dark yellow, bright yellow, white, bright red and reddish. Some individuals with yellow/ bright green lined arms.	Mostly pentagonal	Small, partially covered by tubercles	6.5 mm - 10.6 mm	Short spinelets, three-four pointed homogenously distributed	Indertidal and shallow subtidal of Atlantic coast of South Europe (Galician coast, Portugal, and the whole shallow subtidal of the Mediterranean Sea
Ophiothrix sp. III	Pink, pale pink, purple, beige, white and/ or whitish	Mostly rounded	Wide, partially covered by tubercles and/ or small spinelets	Very variable among localities:<br>From 7.1mm to 18 mm	Spinelets, homogenously distributed but larger than in O. sp II	Deep subtidal (131-310 m depth) of the Cantabrian Sea, coast of Portugal and Alboran Sea

Table S2. Tables of putative *Ophiothrix* species based on the PTP method. **A** Analysis based on *16S* sequences. **B** Analysis based on *COI* sequences. See supplementary Figs S4 and S5.

A

Maximum likelihood partition of PTP – ***16S* fragment**
Species 1 (support = 1.000)<br>AY365165S1_Macrophiothrix
Species 2 (support = 1.000)<br>AY365153_Macrophiothrix
Species 3 (support = 1.000)<br>AY365160_Macrophiothrix
Species 4 (support = 1.000)<br>AY365145S1_Macrophiothrix
Species 5 (support = 1.000)<br>AY365155S1_Macrophiothrix
Species 6 (support = 1.000)<br>AY365161S1_Macrophiothrix
Species 7 (support = 0.485)<br>AY365170S1_Macrophiothrix, AY365174S1_Macrophiothrix
Species 8 (support = 1.000)<br>AY365179_*O. caespitosa*
**Species 9 (support = 1.000)**<br>**AY365182_*O. trilineata***
**Species 10 (support = 0.913)**<br>**(*Ophiothrix* sp. III)**<br>**III_4,III_7,III_18,III_17,III_12,III_9,III_1,III_10,III_16,III_3,III_14,III_5,III_15,III_8,III_13,III_2,III_11,III_19,III_6**
**Species 11 (support = 0.740)**<br>**(*Ophiothrix* sp. II)**<br>**II_18,II_16,II_10,II_55,II_43,II_29,II_30,II_48,II_26,II_21,II_47,II_2,II_50,II_33,II_40,II_7,II_27,II_28,II_14,II_25,II_52,II_19,II_44,II_57,II_3,II_1,II_5,II_51,II_24,II_39,II_34,II_11,II_30_b,II_20,II_8,II_54,II_59,II_45,II_38,II_56,II_9,II_6,II_57_b,II_53,II_49,II_46,II_42,II_41,II_4,II_36_b,II_35,II_32,II_31,II_23,II_15,II_12,II_13**
**Species 12 (support = 0.892)**<br>**(*Ophiothrix fragilis*)**<br>**fra_19,fra_26,fra_22,fra_8,fra_24,fra_25,fra_21,fra_4,fra_28,fra_31,AJ002795_*O.quinquemaculata*,fra_27,fra_6,fra_34,fra_30,fra_29,fra_33,fra_32,fra_7,fra_16,fra_14,fra_15,fra_17,fra_2,fra_1,fra_20,fra_23,fra_3,fra_9,fra_18,fra_12,fra_5,fra_11,fra_10,fra_13**

B

Maximum likelihood partition of PTP – ***COI* fragment**
Species 1 (support = 1.000)<br>AY365151S2_Macrophiothrix_koehleri
Species 2 (support = 1.000)<br>Ophiothrix_suensoni_LK026603
**Species 3 (support = 0.873)**<br>**(*Ophiothrix* sp. II)**<br>**Hap_75,Hap_12,Hap_109,Hap_57,Hap_2,Hap_30,Hap_64,Hap_36,Hap_73,Hap_80,Gal1,Gal2,Gal3,Hap_31,Hap_78,Hap_104,Hap_25,Hap_39,Hap_42,Hap_112,Hap_40,Hap_103,Hap_50,Hap_66,Hap_70,Hap_58,Hap_53,Hap_56,Hap_93,Hap_41,Hap_120,Hap_44,Hap_43,Hap_26,Hap_9,Hap_22,Hap_96,Hap_148,Hap_63,Hap_108,Hap_113,Hap_51,Hap_69,Hap_88,Hap_79,Hap_28,Hap_107,Hap_114,Hap_18,Hap_124,Hap_3,Hap_29,Hap_76,Hap_90,Hap_105,Hap_106,Hap_55,Hap_100,Hap_117,Hap_47,Hap_13,Hap_99,Hap_33,Hap_8,OMed2,OMed1,OMed3,Hap_97,Hap_98,Hap_17,Hap_49,Hap_102,Hap_82,Hap_111,Hap_48,Hap_46,Hap_34,Hap_1,Hap_83,Hap_86,Hap_54,Hap_35,Hap_116,Hap_11,Hap_87,Hap_121,Hap_119,Hap_27,Hap_123,Hap_95,Hap_23,Hap_24,Hap_4,Hap_94,Hap_65,Hap_14,Hap_61,Hap_45,Hap_85,Hap_19,Hap_118,Hap_72,Hap_81,Hap_122,Hap_91,Hap_110,Hap_84,Hap_101,Hap_74,Hap_6,Hap_71,Hap_77,Hap_68,Hap_115,Hap_62,Hap_60,Hap_20,Hap_92,Hap_38,Hap_89,Hap_10,Hap_16,Hap_59,Hap_7,Hap_15,Hap_37,Hap_67,Hap_32,Hap_5,Hap_21,Hap_52**
**Species 4 (support = 0.957)**<br>**(*Ophiothrix fragilis*)**<br>**Hap_138,Hap_127,Hap_125,Hap_131,Ro_JR5_11,Ro_JR5_12,ODig1,Hap_129,Wx_JR6_8,Hap_128,OIE7,Hap_144,Wx_JR6_9,Hap_135,Hap_136,Hap_145,Hap_132,Hap_133,Hap_137,Hap_143,Hap_142,Hap_140,Hap_134,NOR2,NOR1,SU2,SU1,OIE8,Hap_146,ODig10,Hap_126,Hap_141,Hap_147,Hap_139,Hap_130**
**Species 5 (support = 0.959)**<br>**(*Ophiothrix* sp. III)**<br>**III_91.28,III_45.5,III_91.29**
Species 6 (support = 1.000)<br>Ophiothrix_caespitosa_AY365179S2
Species 7 (support = 1.000)<br>Macrophiothrix_longipeda_GU480574
Species 8 (support = 1.000)<br>Ophiothrix_oerstedii_KC626258
Species 9 (support = 1.000)<br>Ophiothrix_aristulata_EU869997
Species 10 (support = 1.000)<br>Ophiothrix_trilineata_GU480577
Species 11 (support = 1.000)<br>Ophiothrix_angulata_KC626245
Species 12 (support = 1.000)<br>Ophiothrix_lineata_EF053424
Species 13 (support = 1.000)<br>Ophiothrix_spiculata_KC626260
Species 14 (support = 1.000)<br>Ophiothrix_accedens_KF663468
Species 15 (support = 1.000)<br>AY365145S2_Macrophiothrix_caenosa
Species 16 (support = 0.994)<br>AY365165S2_Macrophiothrix_megapoma,AY365155S2_Macrophiothrix_leucosticha
Species 17 (support = 1.000)<br>Macrophiothrix_rhabdota_AY365174S2
Species 18 (support = 1.000)<br>AY365161S2_Macrophiothrix_lorioli

Table S3. Pairwise *COI* genetic distances based on a Kimura 2-parameter model (K2p) between *Ophiothrix* species available from Genbank (171 sequences), and sequences obtained in this study. SE, standard error.

Species 1	Species 2	Kp2	SE
Ophiothrix sp. II	*O. fragilis*	0.1895	0.02233
Ophiothrix sp. II	*Ophiothrix* sp. III	0.2200	0.02438
O. fragilis	*Ophiothrix* sp. III	0.2021	0.02307
Ophiothrix sp. II	*O. trilineata*	0.2798	0.02994
O. fragilis	*O. trilineata*	0.2367	0.02640
Ophiothrix sp. III	*O. trilineata*	0.2642	0.02791
Ophiothrix sp. II	*O. caespitosa*	0.2696	0.02970
O. fragilis	*O. caespitosa*	0.2675	0.02996
Ophiothrix sp. III	*O. caespitosa*	0.2540	0.02675
O. trilineata	*O. caespitosa*	0.2932	0.03222
Ophiothrix sp. II	*O. accedens*	0.2652	0.02784
O. fragilis	*O. accedens*	0.2480	0.02626
Ophiothrix sp. III	*O. accedens*	0.2419	0.02665
O. trilineata	*O. accedens*	0.2612	0.02848
O. caespitosa	*O. accedens*	0.2602	0.02920
Ophiothrix sp. II	*O. aristulata*	0.2453	0.02719
O. fragilis	*O. aristulata*	0.2145	0.02482
Ophiothrix sp. III	*O. aristulata*	0.2000	0.02427
O. trilineata	*O. aristulata*	0.2543	0.02818
O. caespitosa	*O. aristulata*	0.2392	0.02757
O. accedens	*O. aristulata*	0.1966	0.02393
Ophiothrix sp. II	*O. angulata*	0.2655	0.02900
O. fragilis	*O. angulata*	0.2495	0.02570
Ophiothrix sp. III	*O. angulata*	0.2698	0.02861
O. trilineata	*O. angulata*	0.2278	0.02480
O. caespitosa	*O. angulata*	0.2543	0.02815
O. accedens	*O. angulata*	0.2376	0.02607
O. aristulata	*O. angulata*	0.2541	0.02825
Ophiothrix sp. II	*O. lineata*	0.2493	0.02711
O. *fragilis*	*O. lineata*	0.2324	0.02588
Ophiothrix sp. III	*O. lineata*	0.2291	0.02526
O. trilineata	*O. lineata*	0.2170	0.02601
O. caespitosa	*O. lineata*	0.2496	0.02898
O. accedens	*O. lineata*	0.2027	0.02434
O. aristulata	*O. lineata*	0.2024	0.02481
O. angulata	*O. lineata*	0.2054	0.02452
Ophiothrix sp. II	*O. spiculata*	0.2621	0.02797
O. fragilis	*O. spiculata*	0.2528	0.02755
Ophiothrix sp. III	*O. spiculata*	0.2307	0.02628
O. trilineata	*O. spiculata*	0.2610	0.02869
O. caespitosa	*O. spiculata*	0.2730	0.03011
O. accedens	*O. spiculata*	0.2158	0.02556
O. aristulata	*O. spiculata*	0.2380	0.02693
O. angulata	*O. spiculata*	0.2888	0.03110
O. lineata	*O. spiculata*	0.1907	0.02349
Ophiothrix sp. II	*O. oerstedii*	0.3017	0.03060
O. fragilis	*O. oerstedii*	0.2928	0.03039
Ophiothrix sp. III	*O. oerstedii*	0.2620	0.02760
O. trilineata	*O. oerstedii*	0.2574	0.02765
O. caespitosa	*O. oerstedii*	0.2481	0.02805
O. accedens	*O. oerstedii*	0.2307	0.02637
O. aristulata	*O. oerstedii*	0.2091	0.02462
O. angulata	*O. oerstedii*	0.2571	0.02773

O. lineata	*O. oerstedii*	0.2055	0.02413
O. spiculata	*O. oerstedii*	0.2543	0.02758
Ophiothrix sp. II	*O. suensoni*	0.2211	0.02482
O. fragilis	*O. suensoni*	0.2455	0.02734
Ophiothrix sp. III	*O. suensoni*	0.2780	0.03053
O. trilineata	*O. suensoni*	0.2852	0.03141
O. caespitosa	*O. suensoni*	0.2377	0.02698
O. accedens	*O. suensoni*	0.2610	0.02807
O. aristulata	*O. suensoni*	0.2442	0.02753
O. angulata	*O. suensoni*	0.2341	0.02605
O. lineata	*O. suensoni*	0.2244	0.02656
O. spiculata	*O. suensoni*	0.2447	0.02740
O. oerstedii	*O. suensoni*	0.2811	0.02914

Table S4. Φ_{ST} values between pairs of populations for *Ophiothrix* sp. II.

	Ophiothrix **sp. II**									
	**FER**	**CAS**	**APA**	**CEB**	**LH**	**XAB**	**RS**	**CAD**	**AL**	**LK**
**CAS**	0.00733	–								
**APA**	-0.01032	-0.01243	–							
**CEB**	0.02940	-0.01116	-0.00130	–						
**LH**	-0.01475	-0.01392	-0.02339	0.00786	–					
**XAB**	0.02698	-0.00757	0.00107	-0.00394	0.00782	–				
**RS**	-0.03315	0.00201	-0.01959	0.02485	-0.02799	0.01439	–			
**CAD**	-0.01551	0.01867	-0.00966	0.03665	-0.01140	0.03495	-0.03020	–		
**AL**	0.01792	-0.00316	0.00462	-0.01065	0.00515	-0.02009	0.00417	0.02509	–	
**LK**	0.00655	0.00681	-0.00348	-0.00487	0.00527	0.00869	-0.01196	0.00947	0.00029	–
**KB**	-0.03132	-0.02778	-0.03494	-0.01534	-0.02392	-0.00243	-0.00529	-0.00819	-0.00596	-0.01196

Table S5. Φ_{ST} values between pairs of populations for *Ophiothrix* sp. III.

***Ophiothrix* sp. III**				
	**PINDAL**	**POR**	**DEM45**	**DEM131**
**POR**	-0.07290	–		
**DEM45**	-0.04366	-0.06191	–	
**DEM131**	-0.04891	-0.04571	-0.0360	–
**DEM91**	-0.03199	-0.01309	-0.01847	-0.02689
